# A Rare Encounter: Unravelling the Enigma of a Bilateral Humerus Shaft Fracture With a Unique Mode of Injury

**DOI:** 10.7759/cureus.48288

**Published:** 2023-11-05

**Authors:** Saksham Goyal, Ratnakar Ambade, Ankit M Jaiswal, Rahul Singh, Kashyap Kanani

**Affiliations:** 1 Department of Orthopedics, Jawaharlal Nehru Medical College, Datta Meghe Institute of Higher Education and Research, Wardha, IND

**Keywords:** nailing, crif, orif, humerus, bilateral

## Abstract

Humerus bone fractures make up 4-6% of all adult fractures, of which proximal humerus shaft fracture is only six percent. Simultaneous occurrences of bilateral humerus shaft fractures are infrequently encountered in clinical practice. Precise statistics regarding these injuries are lacking, with scant documentation in the existing literature concerning the subject matter. These fractures may arise due to convulsions triggered by incidents like an electric shock, epilepsy, alcohol withdrawal, and hypoglycemia, which typically give rise to sudden and excessive muscular contractions. Such fractures usually coincide with dislocations of the shoulder joint. However, in our case, the bilateral humerus shaft fractures were caused by physical injuries despite the individual remaining conscious throughout the ordeal. We present a clinical scenario wherein a 28-year-old male sustained fractures in both humerus shafts as a consequence of a road traffic collision with a unique mode of injury, i.e., both the arms of the patient hitting the trolley of a stationary truck. Radiographic investigation revealed a mid-arm shaft fracture on the right side and a fracture of the proximal one-third of the humerus shaft on the left side. He was managed with closed reduction and internal fixation with intramedullary (CRIF) nailing on the right side, and open reduction and internal fixation (ORIF) with plate osteosynthesis for the left side were done. So this is a compelling rare case of bilateral humerus shaft fracture following high-velocity trauma with a unique mode of injury, treated operatively with satisfactory results on follow-up.

## Introduction

Humerus bone fractures make up 4-6% of all adult fractures, of which proximal humerus shaft fracture is only six percent [1]. Simultaneous occurrences of bilateral humerus shaft fractures are infrequently encountered in clinical practice. Precise statistics regarding these injuries are lacking, with scant documentation in the existing literature concerning the subject matter. These fractures may arise due to convulsions triggered by incidents like an electric shock, epilepsy, alcohol withdrawal, and hypoglycemia, which typically give rise to sudden and excessive muscular contractions [2]. A humerus fracture refers to a break in the upper arm bone that connects the shoulder joint to the elbow joint. This type of fracture can occur at any age and may result from trauma or repetitive stress injuries. Humerus shaft fractures can happen to anyone regardless of age or gender if the right injury occurs, but they are most common in males between 21 and 30 years old and females between 60 and 80 years old. About 60% of these fractures happen in the middle part of the humerus bone [3]. The severity of the fracture can vary, ranging from a minor crack in the bone to a complete break that requires surgical intervention. Symptoms of a humerus fracture may include pain, swelling, bruising, and difficulty moving the affected arm. The prevalence of bilateral humerus shaft fracture is very low as compared to unilateral, thus making it a rare case scenario. Bilateral humerus shaft fractures can also be a result of physical trauma, even when the individual remains conscious [4]. Fractures in the middle part of the upper arm bone (mid-shaft humerus fractures) can cause serious damage to nearby nerves and blood vessels, making it crucial to conduct a thorough examination of the structures such as the median nerve and brachial artery to rule out any neurovascular deficit. Specifically, doctors should pay close attention to the distribution of movement and feeling provided by the radial nerve, as well as the pulse in the radial artery. The management of fractures has evolved significantly, giving equal importance to both surgical and non-surgical treatment. Velpeau bandages, Thomas arm splints, and shoulder siccas are a few of the non-surgical methods which are preferred [5]. Humeral shaft fractures are frequently brought on by falls from elevated heights and are commonly the only injury endured. However, they can also arise from high-impact traumatic events such as car accidents. To make an accurate diagnosis of these fractures, it is essential to gather a comprehensive patient history, be mindful of the potential for concurrent injuries that may not be readily observable, and conduct a thorough examination.

## Case presentation

**Figure 1:**
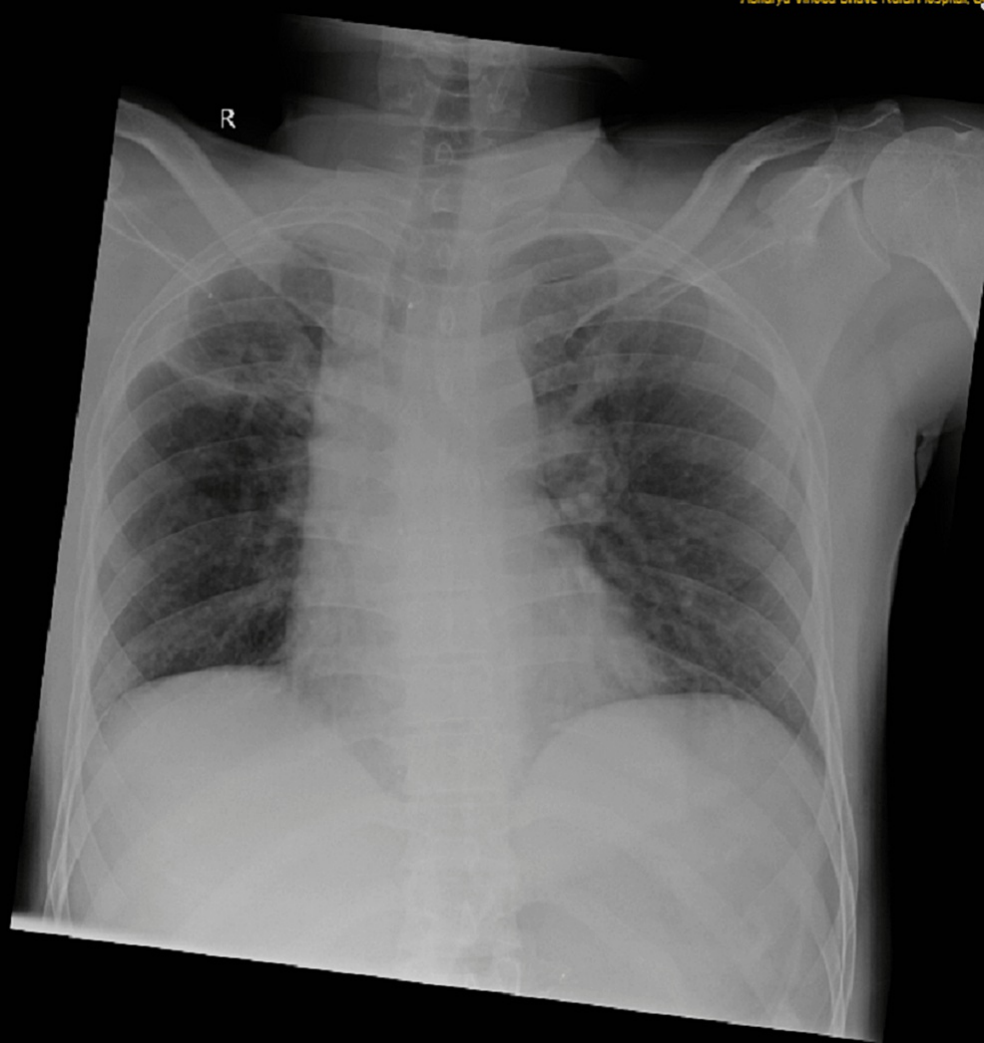
Radiograph of the chest showing no injury

A 27-year-old male was brought to the emergency department of the hospital with presenting symptoms of pain, discomfort, and swelling in both upper arms, along with an incised wound on the forehead. Upon obtaining an extensive medical history, it was revealed that the patient had been involved in a vehicular collision while driving a two-wheeler that collided with a truck trolley a few hours prior. The uniqueness of this incident was that as the patient was driving, his arm came in contact with the trolley, and his chest was spared, as seen in Figure 1. The arms acted as a protective mechanism, thereby preventing the chest injury. The patient complained of excruciating pain in both upper limbs, which was sudden in onset.

**Figure 2:**
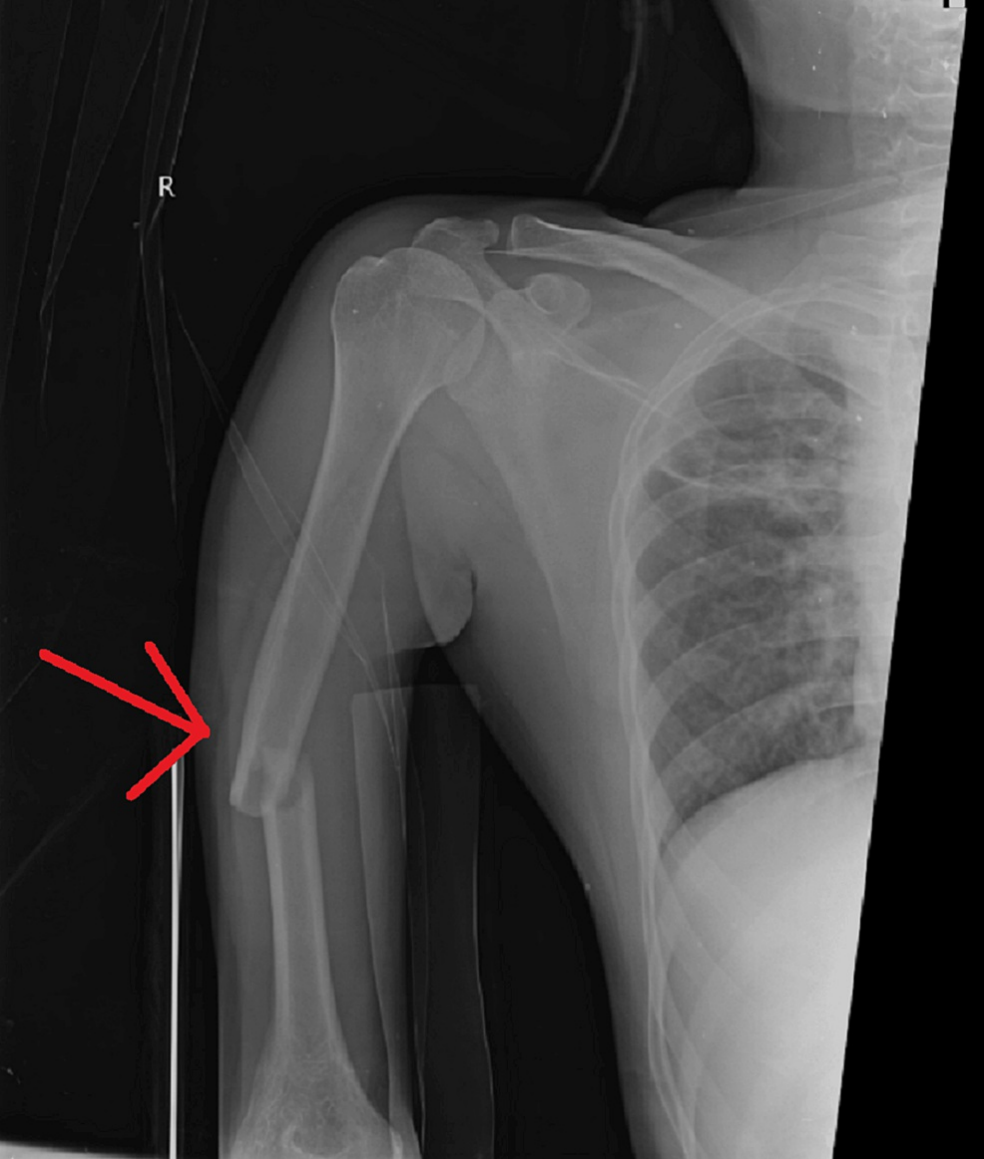
Anterior-posterior radiograph showing short oblique fracture mid-shaft region on right side

**Figure 3:**
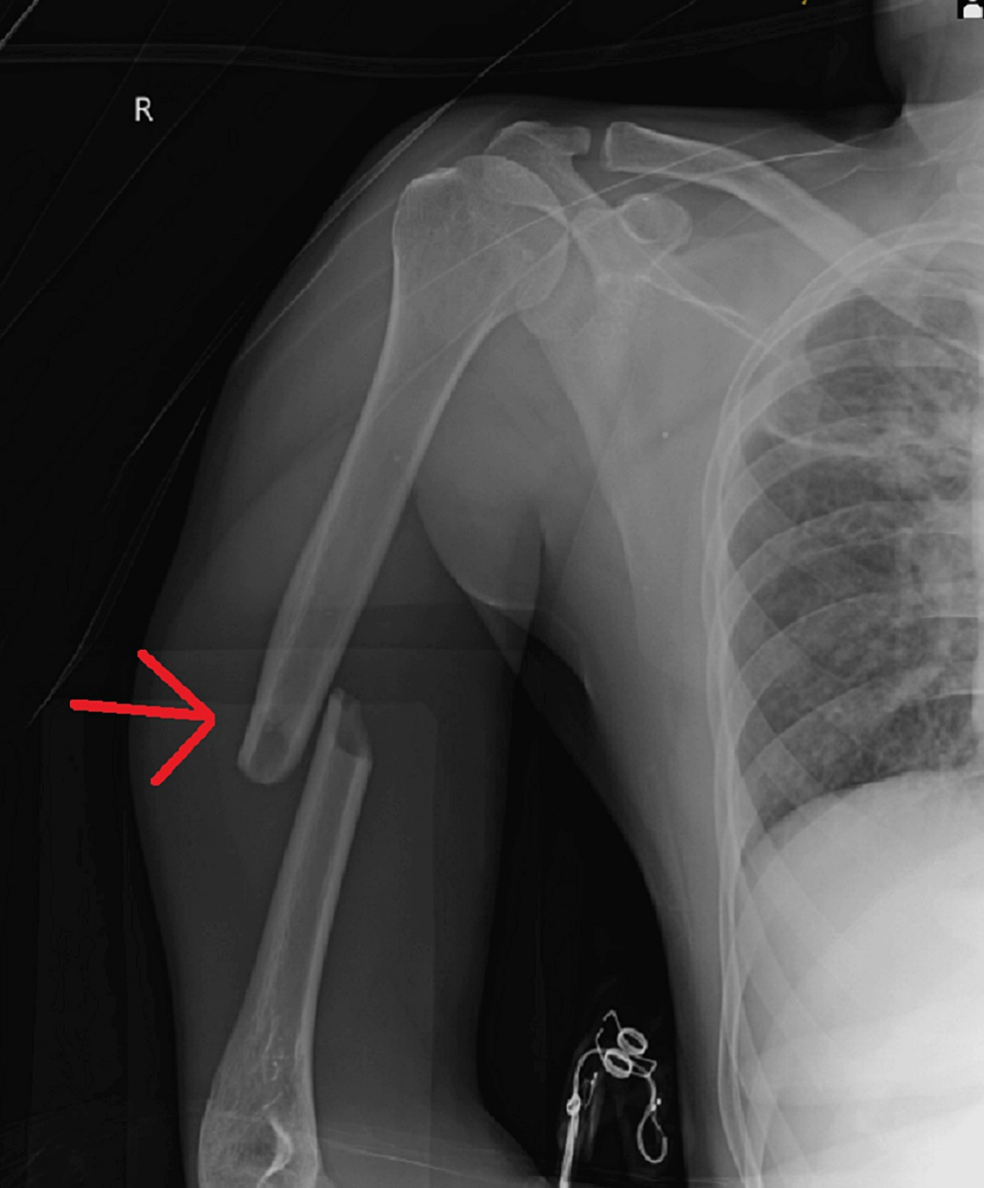
Lateral radiograph showing short oblique fracture mid-shaft region on right side

During the examination of the right upper limb, a visual inspection of the skin surface revealed no signs of scarring or sinus formation. However, there was an observable deformity along the midshaft of the right upper arm, which hindered normal mobility. Additionally, a diffuse swelling was noted in the mid-arm region. Palpation of the midshaft arm of the right upper limb revealed tenderness and crepitus, along with the elevated local temperature. While testing wrist and finger mobility, a full range of motion was noted, although thumb extension appeared to be weakened. No distal neurovascular deficit was noted. Upon palpation, the right arm shaft region exhibited tenderness and swelling, accompanied by bony crepitus. Hand mobility was found to be abnormal, and a mild inflammation was suggested by an increase in local temperature. Assessment of wrist mobility indicated a full range of motion with normal movement. Examination of the fingers revealed normal movement, with a full range of motion, albeit with weakened limb extension. A radiograph of the right upper limb revealed a short oblique fracture in the mid-shaft region on the right side (Figures 2, 3).

**Figure 4:**
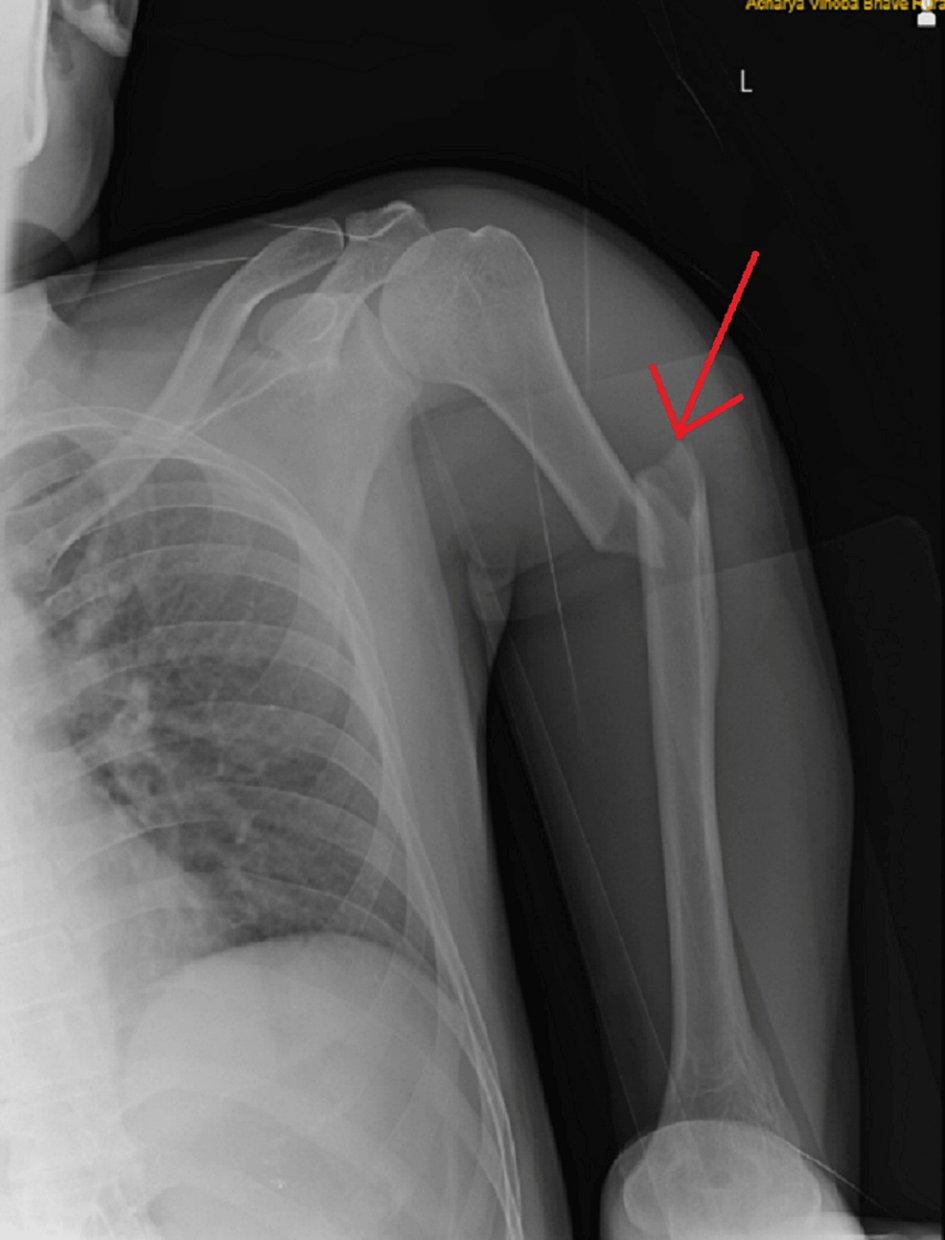
Anterior-posterior radiograph showing short oblique fracture in proximal humeral shaft region on left side

**Figure 5:**
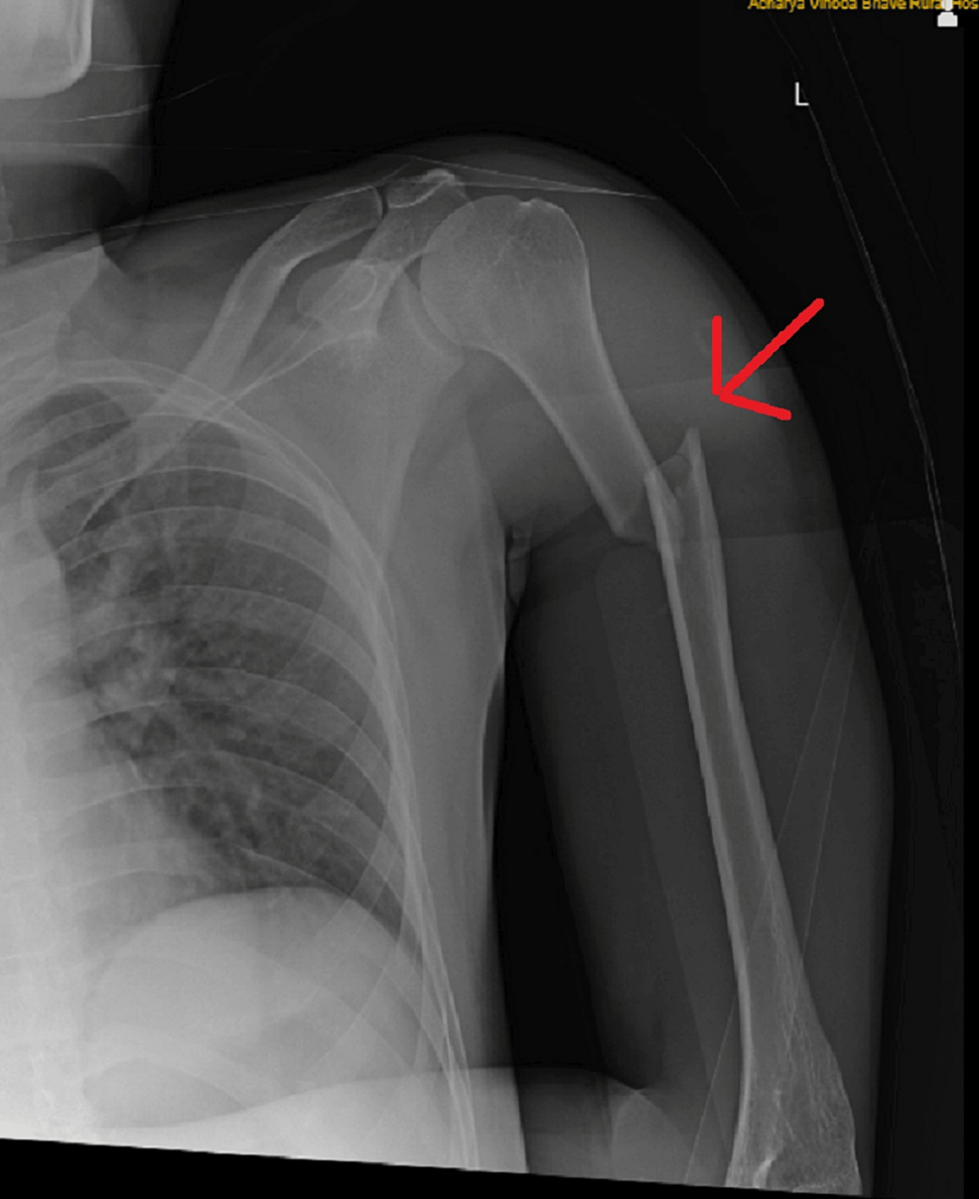
Lateral radiograph showing short oblique fracture in proximal humeral shaft region on left side

On examination of the left upper limb and visual inspection, abrasions were noted on the overlying skin along the anterolateral aspect of the humerus, although no signs of scarring or sinus formation were observed. A deformity was observed along the proximal one-third of the arm, accompanied by swelling in the mid-arm region. Upon palpation of the left arm, the initial observations were confirmed, revealing tenderness and crepitus along the proximal one-third of the shaft, while the radial artery remained palpable. Further examination indicated weakened finger movement, specifically in abduction and opposition, and also weak thumb extension. Additionally, decreased sensation was noted over the ulnar border, along with a tingling sensation present on the left side. A radiograph of the left upper limb revealed a short oblique fracture in the proximal humerus shaft region on the left side (Figures 4, 5).

**Figure 6:**
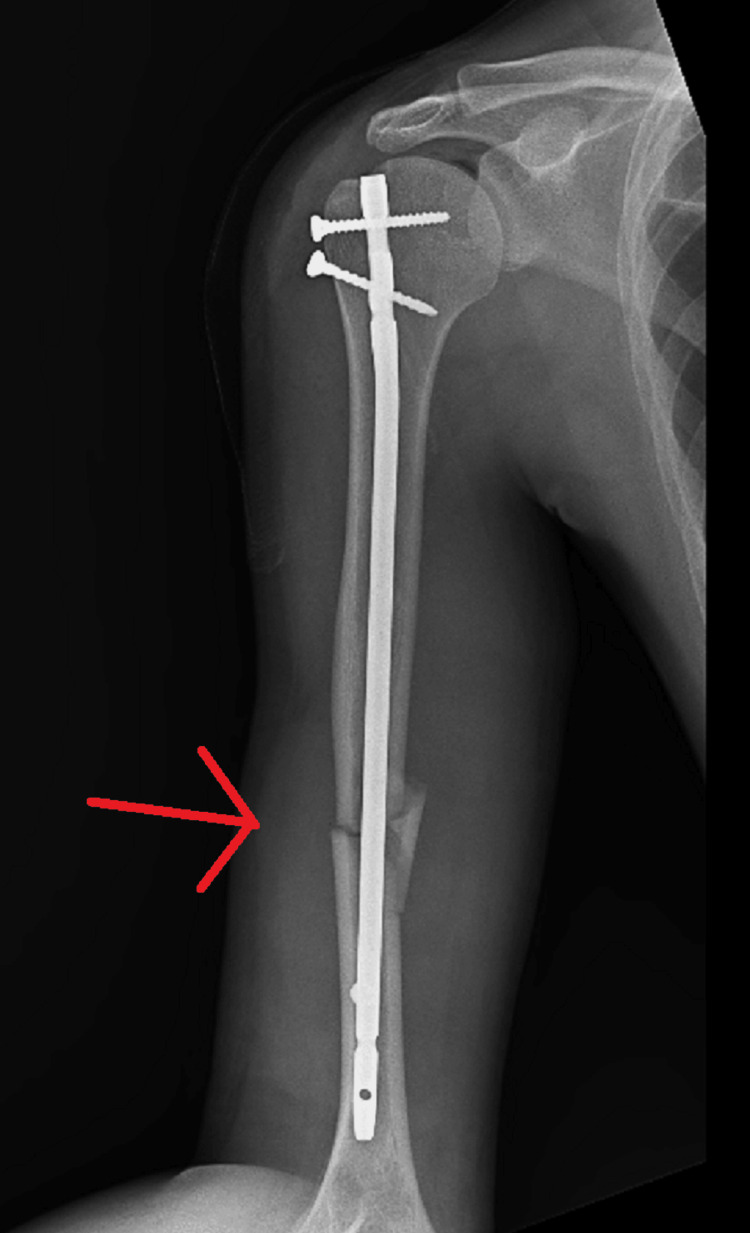
Anterior-posterior radiograph showing CRIF with intramedullary nailing for mid-shaft humerus fracture on the right side CRIF - closed reduction and internal fixation

**Figure 7:**
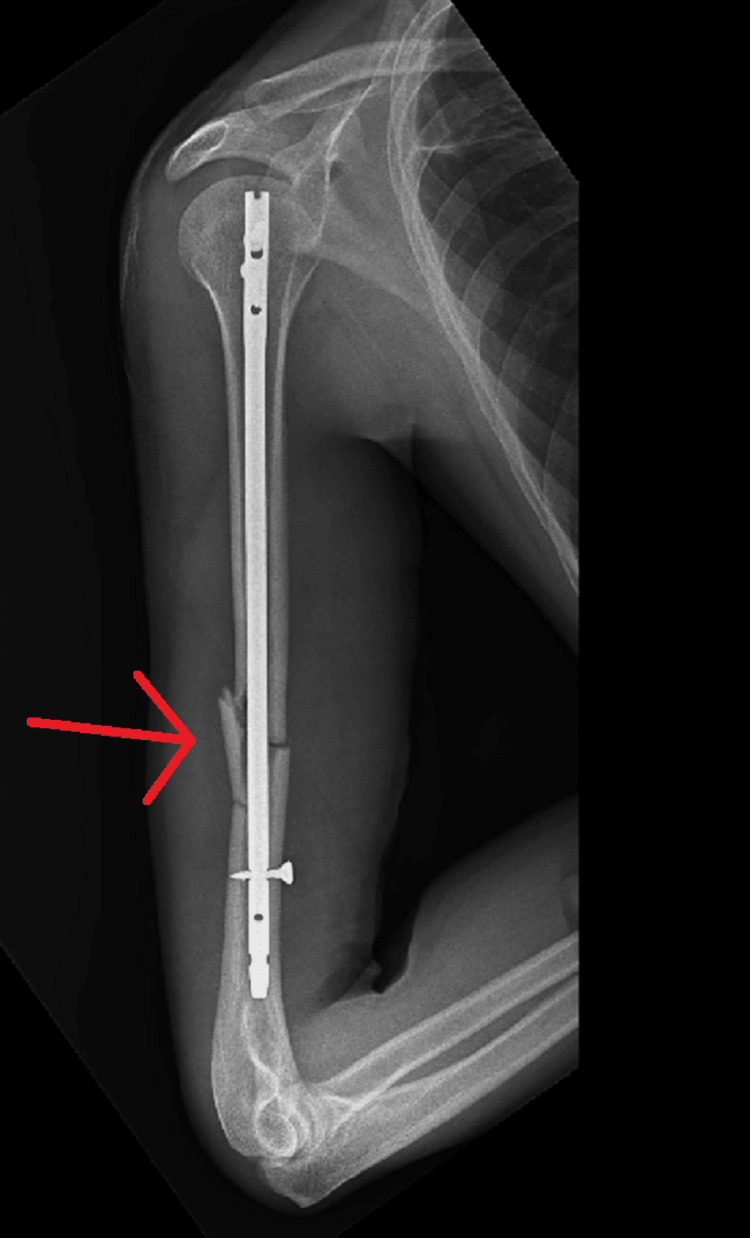
Lateral radiograph showing CRIF with intramedullary nailing for mid-shaft humerus fracture on the right side CRIF - closed reduction and internal fixation

**Figure 8:**
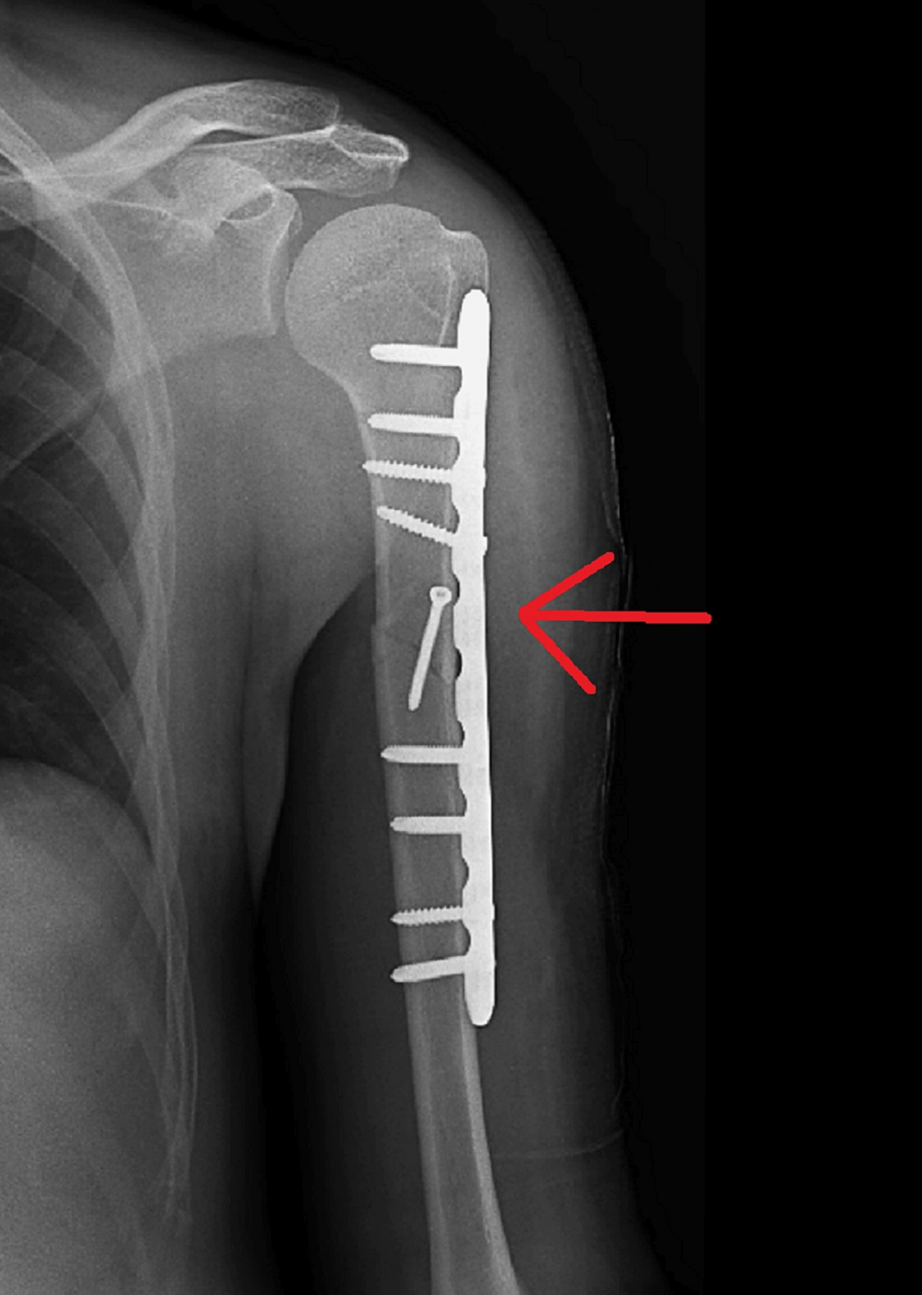
Anterior-posterior radiograph showing ORIF with plate osteosynthesis for proximal one-third humerus shaft fracture on left side ORIF - open reduction and internal fixation

**Figure 9:**
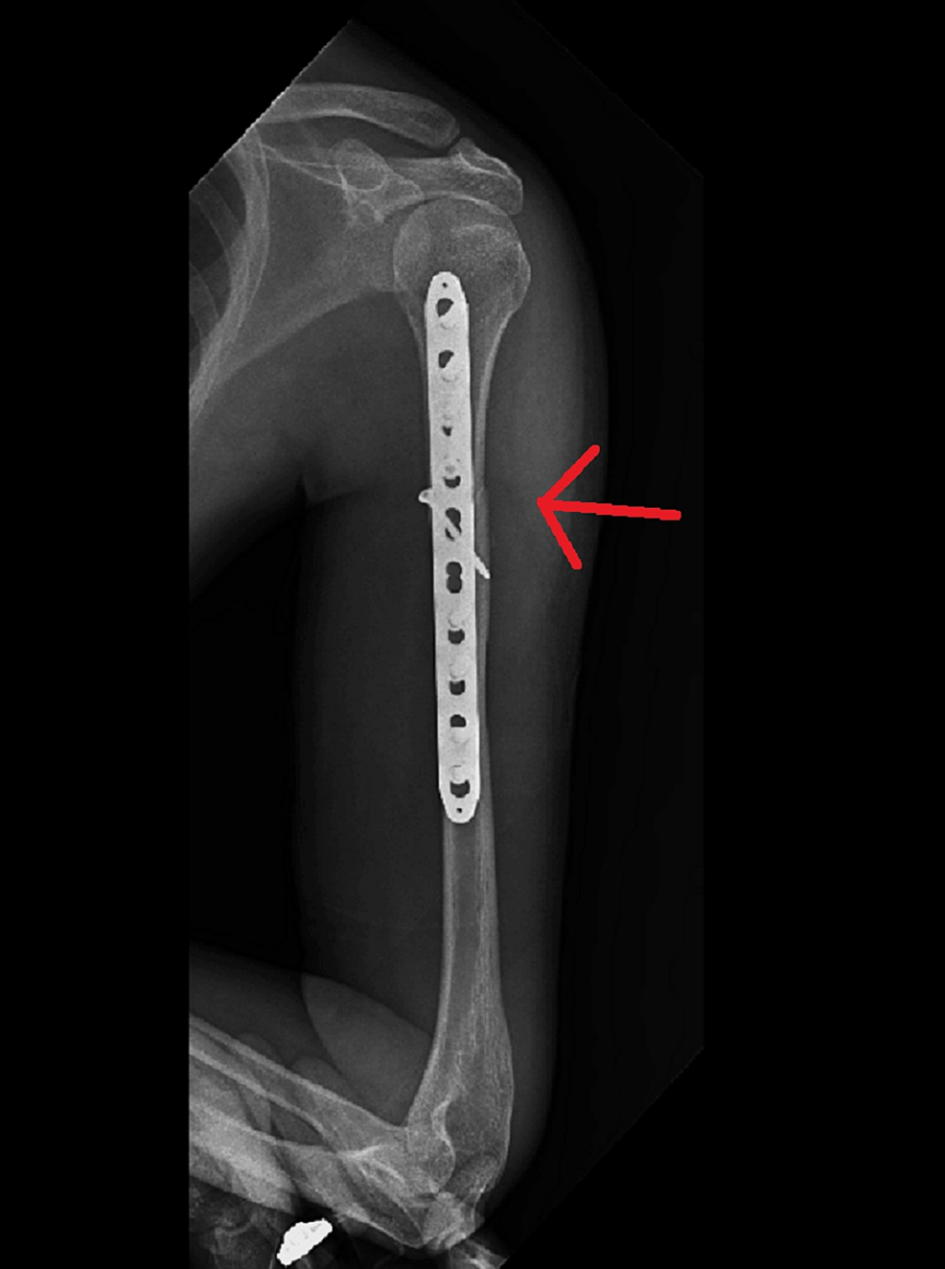
Lateral radiograph showing ORIF with plate osteosynthesis for proximal one-third humerus shaft fracture on left side ORIF - open reduction and internal fixation

The patient was scheduled to undergo general anesthesia for the surgery. Both shoulders were addressed in a single surgical session, with the right side being operated on first, close reduction internal fixation with intramedullary nailing for mid-shaft humerus fracture taking approximately two hours (Figure 6, 7) followed by the left side, as open reduction internal fixation with plate osteosynthesis for proximal one-third humerus shaft which took approximately three hours (Figure 8, 9). Re-draping was undertaken, and before the commencement of the left side, an intravenous antibiotic was administered.

**Figure 10:**
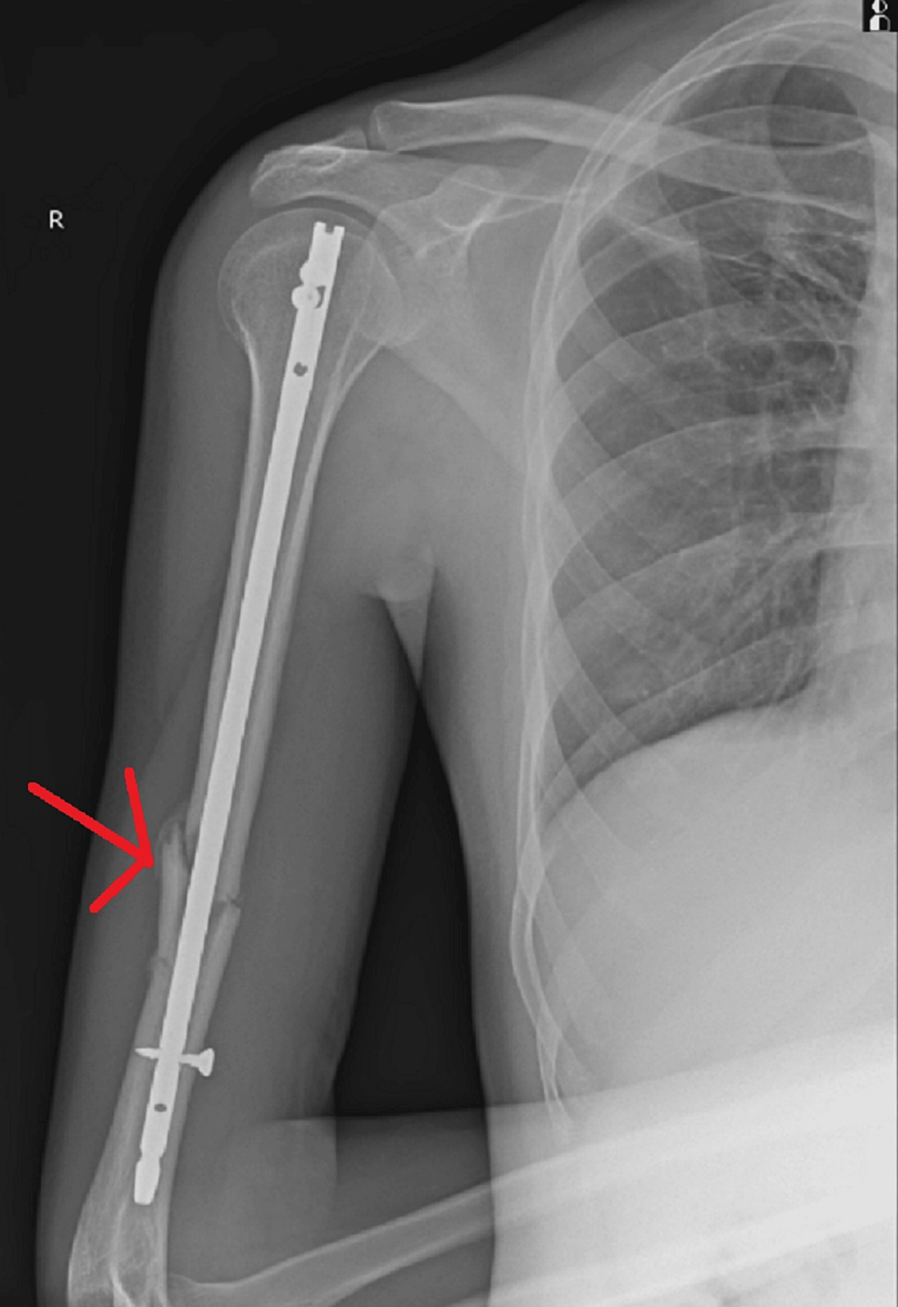
Post-one-month radiograph showing CRIF with intramedullary nailing for mid-shaft humerus fracture on the right side CRIF - closed reduction and internal fixation

**Figure 11:**
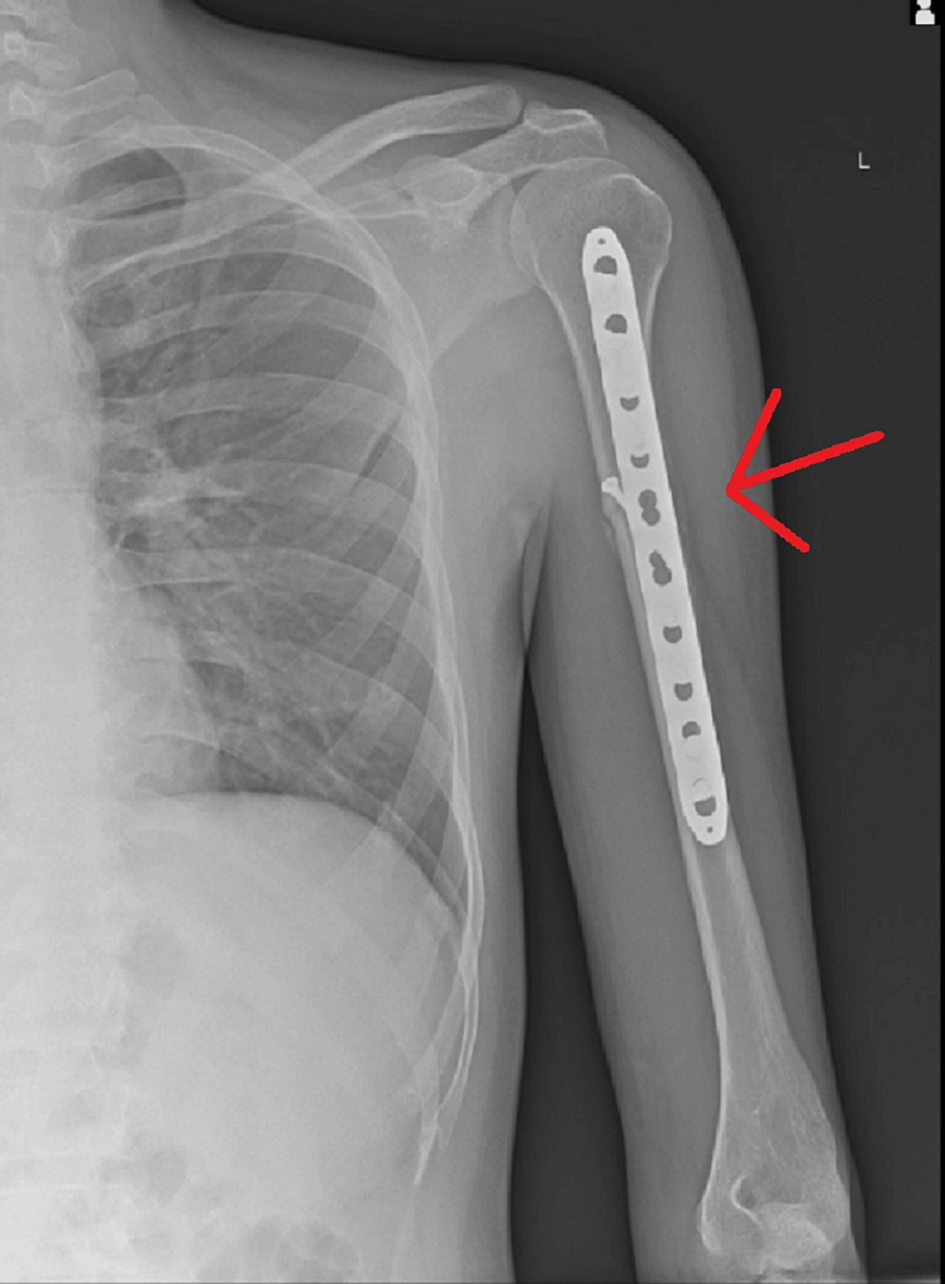
Post-one-month radiograph showing ORIF with plate osteosynthesis for proximal one-third humerus shaft fracture on left side ORIF - open reduction and internal fixation

The patient came for follow-up after one month, and rehabilitation exercises were continued in the form of shoulder pendulum, elbow range of movement, shoulder shrugging exercises, and shoulder abduction as per tolerance on the right side. Rehabilitation exercises in the form of shoulder pendulum, elbow range of movement, and shoulder shrugging exercises were continued on the left side. Follow-up of radiographs after one month are shown in Figure 10 and Figure 11.

## Discussion

Cases, such as the one described here, are rare in the medical literature due to the complex nature of the injury that led to this clinical scenario. The patient sustained an oblique fracture from a direct blow at an angle to an outstretched arm, which is typically associated with chest injuries. However, in this case, the patient's arms were outstretched at the time of the incident, which prevented direct trauma to the chest wall. Due to the infrequency of bilateral humeral shaft fractures, there is currently no established standard of care for their treatment. When determining the appropriate treatment, various factors must be considered, including the shape of the fracture, the patient's overall health, and any other injuries the patient may have. Surgery may be recommended for young adults to avoid complications such as deformity, nonunion, and contraction. Several methods are available to manage humeral shaft fractures, including conservative management, open reduction and internal fixation (ORIF) with a plate, intramedullary nailing (IMN) through closed reduction, and, in rare cases, an external fixator may be used. Proximal humerus fractures are not common among individuals under 50 years old. There are different types of humerus shaft fractures, such as transverse fractures, which can result in nonunion. Oblique and spiral fractures more commonly cause radial nerve palsy as well as mal-union.

In this case, on the right side, we performed open reduction first and then used nails for minimally invasive surgery. One benefit of this approach is the use of nails, which can reduce invasiveness. Also, intramedullary nailing provides relative stability, which has a better outcome and has a lower risk of infection as compared to other surgical techniques, such as open reduction and internal fixation [6]. The incision made in the skin is smaller, and the metal rod is inserted into the medullary canal, reducing the exposure of the bone fragments to bacteria. Intramedullary nailing promotes faster healing of the fractured bone. The metal rod provides a stable environment for the bone fragments to heal, allowing for an early range of motion and early weight-bearing. However, there is a risk of nerve and vascular injury during the closed reduction process [7].

Some authors recommend performing plating surgery before reducing a shoulder dislocation to minimize neurovascular injury. Plate osteosynthesis allows for greater precision in aligning the bone fragments. Additionally, reducing a more chronic dislocation can be more challenging. During surgery, the radial nerve can be observed, but it is unclear whether exposing it in cases where there is no nerve damage is beneficial. Furthermore, plating surgery requires a larger skin incision compared to nailing [8].

Many professionals suggest that splinting before reduction could decrease the risk of neurovascular damage during the procedure and improve the range of motion in elderly patients. However, it is important to consider the potential risks of nonunion, contracture, and prolonged treatment duration for young patients when using this approach [9].

In a case report where the patient had a degloving injury, external fixation was used to treat the shoulder dislocation. After the external fixation was applied, a reduction of the dislocated shoulder was performed. While external fixation can prevent neurovascular injury, it may not be the best option for patients without skin injuries since it can negatively affect rehabilitation and patient convenience [10].

## Conclusions

Proximal humerus shaft fractures are frequently encountered, and there is an ongoing discussion about the best treatment method based on age. However, bilateral proximal humerus shaft fractures are infrequent, resulting from direct trauma to both arms simultaneously, without involving the chest region. In this case, the young patient underwent intramedullary nailing on the right arm and plate osteogenesis on the left arm as treatment modalities. Each case must be assessed because both type of treatment is better in their situation for a certain fracture pattern. The use of plating as opposed to interlocking nails in the treatment of closed humeral shaft fracture has been found to produce better overall outcomes. The plating groups exhibit a propensity for earlier union due to absolute stability.
